# Longitudinal profile of neutralizing and binding antibodies in vaccinated and convalescent COVID‐19 cohorts by chemiluminescent immunoassays

**DOI:** 10.1002/iid3.612

**Published:** 2022-05-19

**Authors:** Xiaowen Dou, Enyun Wang, Ruiwei Jiang, Min Li, Dan Xiong, Baoqing Sun, Xiuming Zhang

**Affiliations:** ^1^ Department of Medical Laboratory The Third Affiliated Hospital of ShenZhen University Shenzhen China; ^2^ Department of Allergy and Clinical Immunology, Guangzhou Institute of Respiratory Health, First Affiliated Hospital of Guangzhou Medical University Guangzhou Medical University Guangzhou Guangdong China; ^3^ Department of Medical Laboratory, Shenzhen Luohu Hospital Group Shenzhen Luohu People's Hospital Shenzhen China

**Keywords:** antibody profile, COVID‐19 convalescent, neutralizing antibody, SARS‐CoV‐2 vaccine, seroconversion rate

## Abstract

**Introduction:**

Surrogate rapid serological assay was urgently demanded for accessibly interpretation of immunity potency and duration of neutralizing antibody against SARS‐CoV‐2. The longitudinal trajectory of antibody profile with a reliable large‐scale assay was crucial to judge the protective immune status, avoid futile therapy and provide insight into the booster vaccination minimizing the risk of COVID‐19.

**Methods:**

A total of 195 volunteers were enrolled for a two‐doses procedure (0 and 28 days) of inactive vaccination, as well as ten COVID‐19 convalescents. The serum was collected at six time point and detected by chemiluminescent immunoassay with SARS‐CoV‐2 neutralizing antibody (Nab), SARS‐CoV‐2 RBD immunoglobulin G (IgG) antibody (RBD IgG) and RBD total antibody. The diagnostic results and the correlation of antibody level were evaluated among three serological (Nab, RBD IgG, and RBD total antibody) assay, as well as with an authorized cPass kit (Nab). Referred to the assay‐specific threshold, the seroconversion rate and dynamic titer of antibody were exhibited from 0 to 56 days since vaccination.

**Results:**

There was no difference observed with diagnostic results between neutralizing and RBD IgG antibody (*p* > 0.05). Both diagnostic results of neutralizing and RBD IgG antibody testing differentiated from RBD total antibody assay (*p* < 0.05). The coefficient of correlation (R) was above 0.90 among the levels of those three antibodies, more than 0.60 in comparison with neutralizing antibody by cPass enzyme‐linked immunoassay. The “S” varying pattern for various antibodies level was observed with time extension after vaccination. The seroconversion rate was below 11.1% in 2 weeks after the priming dose, while the value climbed to 81% in 1 week after the boosting dose. The seroconversion rate was maintained around 91%. The inactive vaccine elicited 81‐fold higher antibody levels after finished the vaccination schedule than that at the basic point. Besides, the level of neutralizing antibody induced by vaccine was found with a 0.2‐fold ratio by comparison with that in COVID‐19 convalescents.

**Conclusion:**

The humoral immune response products including SARS‐CoV‐2 neutralizing, RBD IgG antibody and total antibody and the varying pattern of the antibody profile could be rapidly detected by CILA method. Meanwhile, the continuing and dynamic determination was attributed to evaluate the protection effect of humoral immunity against the SARS‐CoV‐2 infection.

## Graphical abstract

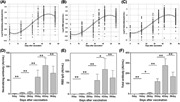

## 1 INTRODUCTION

Since the SARS‐CoV‐2 outbreak, the pandemic has swept the globe and brought on a serious life‐threaten with over 191 million COVID‐19 cases and 4.12 million deaths, as well as a heavy blow to the global economy.^1^ In view of super transmission ability, specific drugs deficiency and frequent mutant strains of SARS‐CoV‐2 occurring, the vaccination against SARS‐CoV‐2 infection and transmission and the disease was expected as a game‐changing tool for ending the COVID‐19 pandemic as soon as possible. Building the colony immune defence through universal vaccination has escalated into a worldwide strategic issue for combating SARS‐CoV‐2. As of 21th July 2021, over 1.4 billion doses of SARS‐CoV‐2 vaccine has been receipted in China from official organization report. Every country is working tirelessly to expedite the vaccination rollout worldwide.

Detection of serum anti‐SARS‐CoV‐2 antibodies such as total antibodies, SARS‐CoV‐2 immunoglobulin G (IgG)/IgM, RBD IgG, and neutralizing antibodies (Nabs) can not only provide an assisted approach for identifying the infection status of the body, but also protect convalescent patients from reinfection with SARS‐CoV‐2. With extensive vaccination, much attention has been paid to study on the persistence of antiviral serum antibodies after vaccination and in convalescent patients. Particularly, protective Nabs present in the body preferentially binding to epitopes (receptor‐binding domain, RBD) in S protein of SARS‐CoV‐2, thereby blocking the invasion of the virus into the angiotensin‐converting enzyme 2 (ACE2) on the surface of human cells.^2^ The classical neutralization test based on live pathogen is accurate, and considered as a golden standard. However, the procedure is complex and risky, and implies a restriction on the large‐scale screening of the Nabs efficacy of a vaccine. Previous studies had reported several strategies for immune response of vaccination or infection using rapid Nabs assays, simulation the mechanism of virus infection.^3^, ^4^, ^5^, ^6^, ^7^ A surrogate virus neutralization test (VNT) with enzyme‐linked immunoassay (ELISA) method (cPass™ sVNT Kit) was developed and authorized for Nabs detection for COVID‐19 cohort. In comparison with the former, the rapid assay achieved with 99.9% specificity and 95%–100% sensitivity was expected to be routine application in clinical and general laboratory.^3^ Padoan et al.^4^ found the SARS‐CoV‐2 RBD IgG assay possessing a moderate agreement (ρ = 0.689) with neutralizing titers (NT). Legros et al.^8^ showed SARS‐CoV‐2 anti‐S IgG titers (Diasorin) worked correlatively with microneutralization Nab titers by Spearman's ρ at 0.7075. Besides, Resman et al.^7^ used an electrochemiluminescence double‐antigen sandwich immunoassay for pan‐anti‐S1‐RBD‐SARS‐CoV‐2 antibodies detection and achieved a less agreement (Cohen's kappa: 0.56) with NTs. Above longitudinal serological and Nabs by rapid assays were mainly focused on COVID‐19 patients, the change pattern was seldom studied on vaccination subjects.^9^ Danese et al.^10^ evaluated the kinetic profile of RBD total antibody, S1/S2 IgG, and RBD IgG antibodies after inoculation of Pfizer messenger RNA (mRNA) vaccine BNT162b2. The three serum antibodies decreased slightly 1 month after reaching the peak on the 35th day, whereas there were only three subjects were included the cohort study. Herein, the characteristic comparison with various serological assays was urgently demanded, so that a large‐scale surveillance for the longitudinal trajectory of antibody profile with COVID‐19 vaccination and recovery was accessible to evaluate the immune response and anticipation of neutralization potency. In this study, except for convalescent patients, a cohort consisting of more than 100 volunteers were enrolled in vaccination group, the profile of the RBD total antibodies, RBD IgG and Nabs were followed up using automatic chemiluminescent immunoassay (CLIA).

## 2 MATERIALS AND METHODS

### 2.1 Patients

Participants were recruited in Shenzhen Luohu People's Hospital from December 2020 to February 2021. The vaccination group aged at 18 and 59 was confirmed by PCR detection and an epidemiology history within 14 days. The participants were included and received a schedule with two‐dose inactivated SARS‐CoV‐2 vaccine. On a voluntary basis, a 2‐month visit was performed with serological detection. Exclusion criteria was following, (1) only one vaccination was received; (2) the interval between two dose was less than 4 weeks. A total of 195 participants were enrolled, including 86 males and 109 females with an average age of 39 years. Among them, 37 cases completed a 2‐month follow‐up, 158 and 122 participants were included for serological analysis at days 14 and 28 after finished the whole vaccination procedure, respectively. Moreover, 4 males and 6 females of the convalescent patients were included with clinical diagnosis and consecutive PCR results. The study was obtained written informed consent and approved by the Ethics Committee of Shenzhen Luohu People's Hospital (2020‐LHQRMYY‐KYLL‐033).

### 2.2 Study design and procedure

Volunteers were inoculated with the inactivated SARS‐CoV‐2 vaccine provided by China National Pharmaceutical Group Co., Ltd. (Sinopharm) through intramuscular injection of the left upper arm. Two doses were given at an interval of about 28 days. In a single‐center, randomized, double‐blind trial, venous blood (3 ml) was collected with a six‐visit, including before the first dose as the baseline sample (recorded as 0 day), 14 and 28 days after first‐dose (14 and 28 days), and 7, 14, and 28 days after the second vaccination (35, 42, and 56 days), respectively. The serum was separated by 3000 r·min^−1^ for 5 min, and was stored at −80°C until serological assays.

### 2.3 Reagents and methods

Three SARS‐CoV‐2 specific antibodies including Nabs, RBD IgG and total RBD antibody were performed on automatic chemiluminescence immunoanalyzer (Mindray CL‐6000i). The ancillary antibody kits (Lot. 2021010100, 2021010100, 2021010400), calibrator and quality control substance were provided by Shenzhen Mindray Biomedical Electronics Co., Ltd. All the testing processes were strictly operated in accordance with the instructions, and the sample volume was 10 μl. As the Manufacturer's interpretation, the result above 10 AU·ml^−1^ was defined as a positive case in serological test, conversely, was a negative sample. Each SARS‐CoV‐2 specific antibody was detected as the following principle.

Nabs detection is based on competitive CLIA. The diluted serum was firstly incubated with recombinant RBD antigen coated with magnetic beads at 37°C. SARS‐CoV‐2 Nabs present in the sample specifically bound to the recombinant RBD antigen on the surface of magnetic beads. Then, the above mixture was incubated with ALP‐ACE2 complex at 37°C, and Nabs competed with ALP‐ACE2 to bind recombinant RBD antigen. Excessive reagent was discarded, the reaction substrate AMPPD was added. The luminous value of CLIA method was inversely proportional to the content of Nabs in the sample, and its content was calculated. RBD total antibody was detected by a double antigen sandwich method on the CLIA platform. Different from Nab analysis, the detection of RBD total antibody applied an ALP‐RBD complex, accordingly, the luminous value was directly proportional to the RBD total antibody content. An indirect CLIA was used for RBD IgG antibody detection. Instead of ALP‐ACE2 complex, the mouse anti‐human IgG labeled with ALP was added and captured the specific IgG antibody fraction.

Reference reagent cPass, SARS‐CoV‐2 Nabs detection reagent is based on the competitive ELISA (A210102; GenScript Biotech Corp, Lot.). In strict accordance with the instructions, the serum (100 μl) was diluted with buffer at 1:9, afterward, the diluted sample was incubated with the same proportion of HRP‐RBD solution at 37°C for 30 min. Then, 100 μl of mixture was added to the capture plate (precoated with hACE2 protein) and incubated at 37°C for 15 min. Finally, the plate was washed with cleaning solution for four times, and the absorbance was recorded at 450 nm after adding TMB solution. Positive and negative quality control were prepared in parallel for inhibition rate. The cutoff with an inhibition rate ≥30% was determined as SARS‐CoV‐2 Nabs positive one.

### 2.4 Statistical analyses

The results were statistical analysis by SPSS 23.0, GraphPad Prism 7.0 and Python. The seroconversion rate was calculated at six‐visit point. McNemar test and paired Wilcoxon test were applied to analyze the differences between groups as well as paired samples, respectively. A *p* < .05 was considered to be statistically significant. Passing‐Bablock regression was used to calculate the correlation between the two methods. In view of magnitude discrepancy between ELISA and CLIA, the data was standardized processing with its cutoff value before the Spearman correlation coefficient analysis.

## 3 RESULT

### 3.1 Diagnosis characteristic comparison between SARS‐CoV‐2 antibody and reference reagent

The seroconversion rates of three SARS‐CoV‐2 antibodies at six‐visit were summarized in Table 1. The visit schedule for serological detection was adapted to the immune response of inactive SARS‐CoV‐2 vaccine. Three serological strategies with paired‐samples was conducted and the results for characteristic comparison was shown in Table 2. The diagnostic result was no significant difference observed between Nabs and RBD IgG (*p* = .70 > 0.05). Whereas, the diagnostic results of RBD total antibody differed from that of Nabs and RBD IgG (*p* = .02, *p* = .01 < 0.05). In comparison with Nabs by cPass, RBD total antibody showed no difference (*p* = .19). On the whole, the assessment of Nabs, RBD IgG and RBD total antibody reached a good agreement with each other (Cohen's Kappa not <0.81). For antibody titer, Nabs, RBD IgG and RBD total antibody by CLIA mutually obtained an wonderful correlation, while a sighter week correlation with Nab by cPass (ELISA, *R* > 0.60).

**Table 1:** Seroconversion rate of antibody profiles after vaccination calculated with each cutoff (10 AU·ml^−1^ for Nabs, RBD IgG, RBD total antibody on Mindray and an inhibition rate ≥30% for Nabs on cPass)

Procedure	Time point	*n*	RBD total Ab	RBD IgG Ab	Neutralizing Ab by mindray	Neutralizing Ab by cPass
Before vaccination	0 day	64	0%	0%	0%	–
The first dose	14 days	54	7.40%	9.26%	11.1%	–
	28 days	51	17.6%	29.4%	23.5%	–
The second dose	35 days	49	83.7%	81.6%	83.7%	–
	42 days	158	91.1%	93.5%	92.4%	82.9%
	56 days	122	83.6%	93.4%	91.8%	86.1%

*Note*: – = represents undetected.

Abbreviations: IgG, immunoglobulin G; Nab, neutralizing antibody.

**Table 2:** Spearman correlation analysis with Passing‐Bablock nonparametric regression method was calculated with paired samples among antibodies by CLIA and ELISA

Item	Clinical performance claimed by manufacture^a^		Total Ab	RBD IgG Ab	Nab by mindray	Nab by cPass
	Sensitivity	Specificity				
Total Ab	100.00% (95% CI: 97.27%–100%)	100.00% (95% CI: 97.27%–100%)	–	0.92 (95% CI: 0.90–0.93) *p* < 0.0001	0.90 (95% CI: 0.89–0.92) *p* < .0001	0.60 (95% CI: 0.52–0.67) *p* < .0001
RBD IgG Ab	99.64% (95% CI: 98.94%–99.88%)	99.64% (95% CI: 98.94%–99.88%)	*p* ^a^ = .01 Kappa 0.81 (95% CI: 0.76–0.86)	–	0.93 (95% CI: 0.92–0.94) *p* < .0001	0.73 (95% CI: 0.67–0.78) *p* < .0001
Nab by mindray	100% (95% CI: 91.24%–100%)	100% (95% CI: 91.24%–100%)	*p* ^a^ = .02 Kappa 0.82 (95% CI: 0.77–0.87)	*p* ^a^ = .701 Kappa 0.88 (95% CI: 0.84–0.93)	–	0.73 (95% CI: 0.67–0.78) *p* < .0001
Nab by cPass	98.9%	100%	*P* ^a^ = .19 Kappa 0.29 (95% CI: 0.14–0.44)	*p* ^a^ = .00 Kappa 0.33 (95% CI: 0.18–0.49)	*p* ^a^ = .00 Kappa 0.32 (95% CI: 0.17–0.48)	–

*Note*: McNemar test and Cohen's kappa statistics also was performed by GraphPad Prism 7.0; – = represents undetected; “a” (with *p* value) represents McNemar test result.

Abbreviations: CI, confidence interval; CLIA, chemiluminescent immunoassay; ELISA, enzyme‐linked immunoassay; IgG, immunoglobulin G.

a The data derived from manufacture's interpretation.

### 3.2 Longitudinal tracking antibody profiles over vaccination time

**Figure 1:**
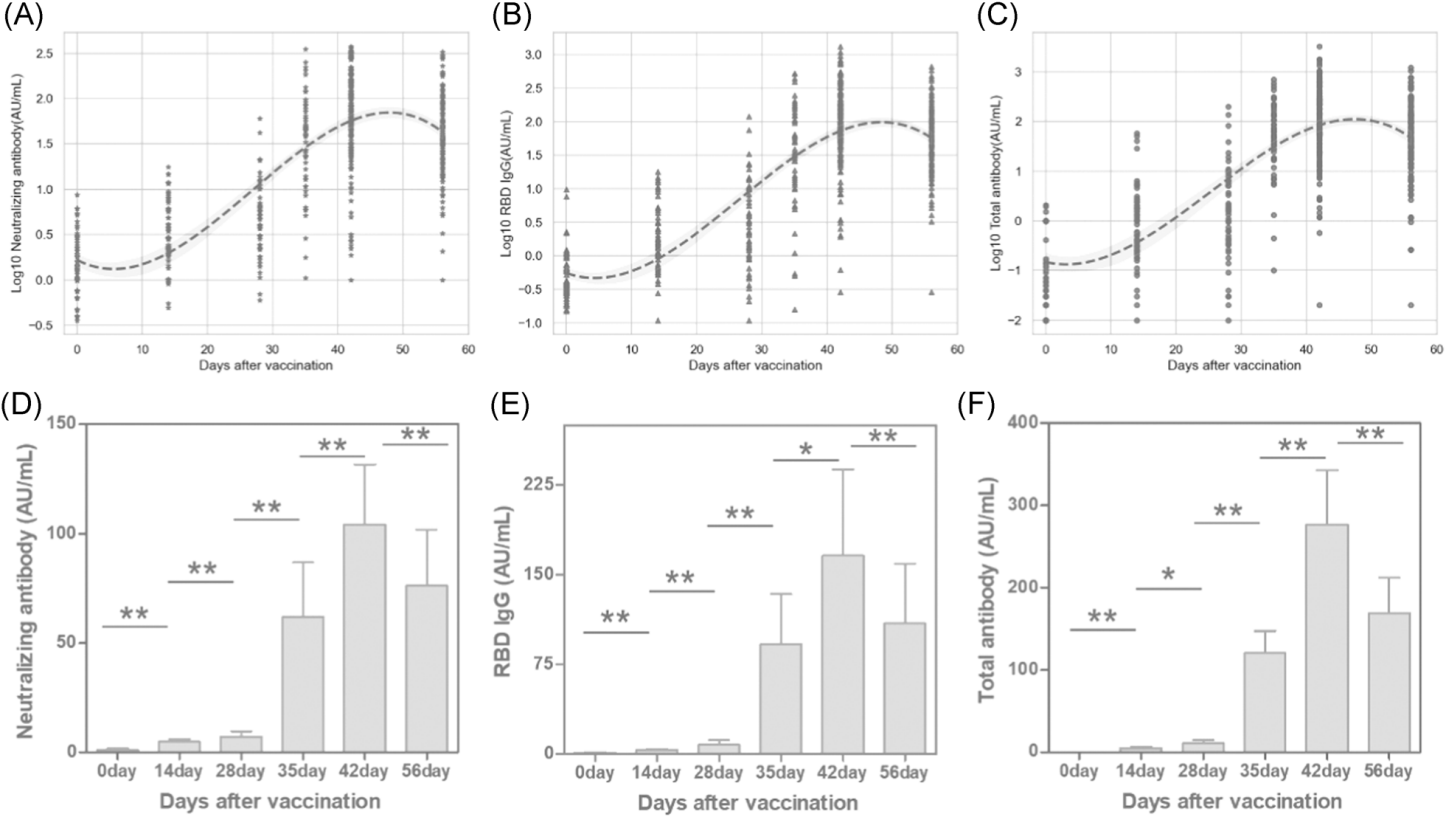
The longitudinal monitoring of neutralizing and binding antibodies RBD IgG and total antibodies titer in vaccinated cohorts with six visits. (A–C) included a total of 195 individuals and were plotted by Python. Among 195 participants, 37 individuals were involved in the whole observation period and the antibodies titers were compared with adjacent visit point by Wilcoxon's matched‐pairs signed‐rank test. *p > .05, **p < .05 (D–F). IgG, immunoglobulin G

As shown in Table 1, all of the baseline for Nabs, RBD IgG and RBD total antibody were negative. Over a 2‐week observation, the seroconversion rate increased to 11.1% Nabs, 9.26% RBD IgG and 7.40% RBD total antibody, respectively. At the third visit, the positive percentage had grown by twofold to threefold. A booster later, the seroconversion rate for each antibody sharply elevated to more than 81% and kept growing with time. Through the 2‐month visit, the recipient achieved the highest seroconversion beyond 93.5% during 14 days postvaccination. Besides, three antibodies titers against inactivated antigens varying with time was displayed in Figure 1A–C. All of antibody titers with time were found exhibition of a “S” Curve through a multivariate regression model. In concert with the seroconversion variation, a rapid ascent in antibody was observed after the second booster immunization. Without the collection point, the highest titer production between 14 and 28 days after the booster vaccination was inferred on, then decreased slightly. The dynamic follow‐up of antibodies titer in the 37 participants were conducted with a paired Wilcoxon test (Figure 1D,E). The humoral immune response induced by vaccine were found with a significant variation after 1–2‐week interval (*p* < .05), especially for Nab level. Compared with the baseline value of 1.272 AU/ml (95% confidence interval [CI]: 0.832–1.712 AU/ml), Nabs levels were increased by threefold and sixfold at 14 and 28 days postvaccination, and rapidly were grown to 61.81 AU/ml (95% CI: 36.94–86.68 AU/ml) after a booster immunization. The maximum 81‐fold increase to 104.1 AU/ml (95% CI: 76.54–131.6 AU/ml) was monitored at 14 days after the second vaccination.

### 3.3 SARS‐CoV‐2 antibody profile in convalescent patients

**Figure 2:**
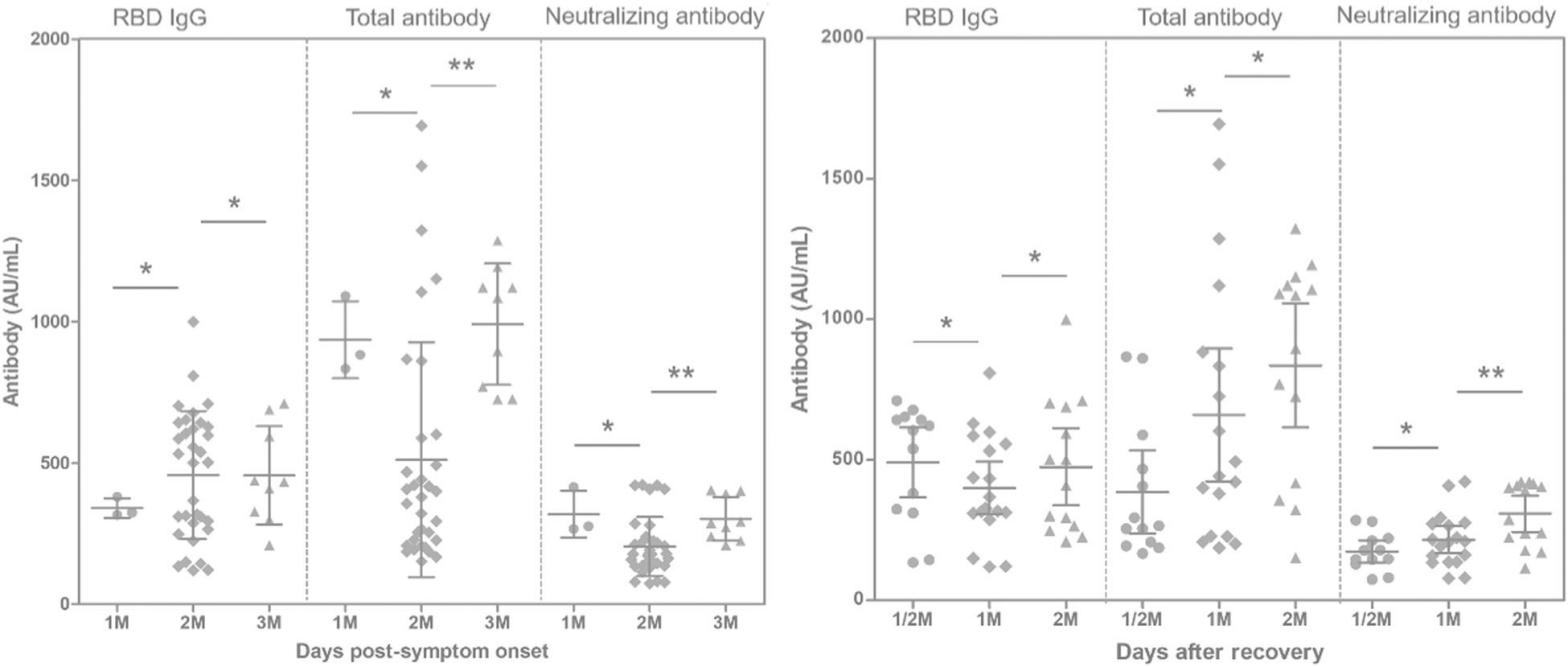
The Nabs, RBD IgG and RBD total antibody titers in convalescent patients changed with the time of onset and recovery time. A Mann–Whitney *U* test were run to determine the difference between adjacent visit point by SPSS 23. The visit point 1, 2, and 3 M represent the patients were detected with antibodies after <15 days, 15–30 days, 30–60 days, and >60 days postsymptom onset and postrecovery, respectively. *p > .05, **p < .05. IgG, immunoglobulin G

The seropositive rates of RBD total antibody, RBD IgG and Nabs in all serum samples (*n* = 46) from convalescent patients were 100%. The antibodies titer changes in ten participants were plotted according to subgroups with the time of onset and recovery time, as shown in Figure 2. The levels of total antibody and Nabs at 1 month postsymptom onset were remarkably greater than 2 months. The protective immune response (Nab) significantly increased during the recovery time. Comparison between the vaccine and COVID‐19 rehabilitation group, the Nabs level induced by the former was 104.1 AU/ml (95% CI: 76.54–131.6 AU/ml), which was 0.2 times higher than that of convalescent patients 511.5 AU/ml (95% CI: 364.2–658.9 AU/ml).

## 4 CONCLUSION

Emerging infections continued spreading, even worse, the existence of delta variant in discovered strains was identified the most transmissibility. However, according to the data with 3.6 billion doses of SARS‐CoV‐2 vaccine administrated until 25th July, 2021, the limited vaccination ratio around 50% proved the protective herd immunity still required a long way off. The Nab titer in COVID‐19 convalescent patients was progressively drop over recovery time in numerous studies, indicating the booster again would probably be proposed for the risk of epidemic and infection. Hence, a commercial rapid serological was in high demand for the large‐scale immune status.

In the study, Nab and binding antibodies including RBD IgG and total antibody were analyzed on an automatic CLIA platform. Lacking of VNT as a standard, Nabs in cPass (ELISA method) authorized by Food and Drug Administration with an emergency schedule was applied as a reference. As expected, Nabs, RBD IgG, and RBD total antibody analysis on CLIA obtained an excellent negative and positive agreement with the reference kit by 99.2%, 98.5%, and 98.5%, respectively. For titer observation, the correlation of RBD IgG and Nabs (*R* = 0.73) with cPass was found a slightly better than that of total antibody (*R* = 0.60). Passive immunity evoked by the inactivated PiCoVacc were similar to those produced by COVID‐19 convalescent patients, RBD antibodies predominated and the key neutralization domain (CND) in RBD structure was found inducing a strong and extensive neutralization response.^11^, ^12^ Padoan et al.^4^ reported a moderate correlation (*R* = 0.689) between Nabs titer with the plaque reduction neutralization test (PRNT_50_) and SARS‐CoV‐2 RBD IgG titer (Snibe diagnostics) with ELISA in 218 convalescent patients. The RBD total antibody detected by a double antibody sandwich method on electrochemiluminescence platform related to the Nabs by PRNT_50_, with an agreement at 0.56 of Cohen' kappa.^7^ A little poor agreement was found between Nabs, RBD IgG, RBD total antibody analysis on an automatic CLIA platform and Nabs on ELISA. The different seroconversion rate at 14 days postvaccination procedure suggested it probably due to the cutoff of cPass derived from COVID‐19 patients. In view of CLIA validated with vaccinated donors by PRNT_50_ in development phase, the characteristics comparison indicated RBD IgG, total antibody ad Nabs on the rapid, automatic and high‐throughput CLIA may be expected to be used an alternative tool of PRNT_50_ for large‐screening the ability of SARS‐CoV‐2 clearance, the risk of reinfection and the passive immune response of vaccine.

After finishing the vaccination procedure, the Nabs seroconversion rate at 92.4% implied biological differences evoked peak immune response existed in individuals and a longer observation required. The previous study with inactivated vaccine BBV152 in phase I clinical trials showed S1 IgG, RBD IgG, and N IgG levels increased rapidly, and the Nabs seroconversion rate was 82.8%–91.9% after two‐vaccination procedure.^13^ CoronaVac vaccine with a 28 days interval program achieved the rates from 79% to 83% and from 92% to 98% giving different doses.^14^ Besides, repeated doses of BBIBP‐CorV caused the Nabs titer to go progressively higher than the single dose.^15^ Similar to above reports, the seroconversion rate was growing steadily from 23.5% at 28 days post the first dose to 91.8% at 28 days after booster immunization. The levels of Nabs, RBD IgG, and RBD total antibody also increased significantly. Hence, strengthening immunity proved to be very important to improve the antibody titer, promote immune response and produce effective protective barrier.

At the visit period, all convalescent patients were detected with 100% seropositive rate, and there was no significant change in each antibody level. Long et al.^16^ pointed out Nabs titer decreased in 81.1 percent of COVID‐19 convalescent patients at the second month after discharge. A 3‐month follow‐up of 164 COVID‐19 patients showed the percentage of 88% individuals produced Nabs and most gradually declined over time.^17^ with the time of rehabilitation. The duration of Nabs may range from 40 days to many years. Vaccine‐induced antibody levels still required a long‐term follow‐up monitoring to assess whether or not enhanced immunization needed.

In accordance with literature before, the Nabs titer induced by vaccine was lower than that in convalescent patients. BNT162B1 vaccine (mRNA) stimulated the body producing immune response by encoding SARS‐CoV‐2 S‐RBD protein. The Nabs titer and RBD IgG level increased rapidly at 7 days but then decreased at 21 days after booster immunization. Besides, in comparison with convalescent patients, the Nabs titer through passive immunity was 0.7–3.5 times, and RBD IgG was 6.5–30 times.^18^ SARS‐CoV‐2 variants were always emerging, Nabs has been proved as cocktail antibody targeting at multiple epitopes, and current reports mainly focused on RBD epitope.^19^, ^20^, ^21^, ^22^ Except for RBD Nabs, non‐RBD Nabs also were screened for blocking SARS‐CoV‐2 entering host through S1‐NTD, S1/S2 epitope, as well as other pathways,^20^, ^23^ indicating the possible presence of unknown binding sites. The limitation in the study is the recombinant RBD antigen expressed by genetic engineering used for assessing the Nabs, which may be not enough to represent the fully protective antibody profile in human.

Plasma therapy with COVID‐19 convalescent patients worked through a high concentration of specific antibody in plasma to neutralize SARS‐CoV‐2, activate complements, and produce an effective immune response. Hence, the antibodies at adequate level laid a foundation for plague prevention and treatment. Equally, the vaccine induced to produce abundant Nabs, which effectively reduced the infection and severe rate. Herein, an automatic CLIA platform were verified to quickly evaluate Nabs, RBD IgG, and total antibody in humoral immune response for vaccine donors and convalescent patients. Moreover, the longitudinal profile of neutralizing and binding antibodies was monitored, and laid a foundation for illustrating the production rules. The study may be helpful to understand how immunity responses against SARS‐CoV‐2 and to offer deep insight into vaccination and therapy for COVID‐19.

## AUTHOR CONTRIBUTIONS

**Xiaowen Dou**: conceptualization, writing‐original draft, software. **Enyun Wang**: methodology, validation. **Ruiwei Jiang**: Fromal analysis, Validation. **Min Li**: software, data curation. **Dan Xiong**: investigation, resources. **Baoqing Sun**: writing‐review and editing. **Xiuming Zhang**: supervision, project administration.

## CONFLICTS OF INTEREST

The authors declare no conflicts of interest.

## Data Availability

The datasets generated and/or analyzed during the current study are not publicly available due to the institute policy to code and archive data in a central repository of the hospital, but are available from the corresponding author on a reasonable request.
